# Supplementary material for: Complementary methods for SARS-CoV-2 diagnosis in times of material shortage
Source: Sci Rep. 2021 Jun 7;11:11899. doi: 10.1038/s41598-021-91457-z (PMC8185005; doi:10.1038/s41598-021-91457-z)
Supplement: Supplementary file 1 — Supplementary Information. [file 41598_2021_91457_MOESM1_ESM.pdf]

Complementary methods for SARS-CoV-2 diagnosis in times of material shortage

Thaisa Lucas Sandri[1,2,3], Juliana Inoue[1], Johanna Geiger[2], Johanna-Marie Griesbaum[1], Constanze Heinzel[1], Michael Burnet[2], Rolf Fendel[1,4], Peter G. Kremsner[1,4,5], Jana Held[1,4,+], Andrea Kreidenweiss[1,4,+,*]

Supplementary information

Supplementary figure S1. Melting curves analysis displaying the different melting temperatures (Tm) of intercalating dye-based assays. **a.** single specific melting peak at 84.5 ºC for hRNAse P gene assay; **b.** single specific melting peak at 80.6 ºC for SARS-CoV-2 RdRp gene assay; **c.** specific melting peak at 81.1 ºC for SARS-CoV-2 E gene; **d.** differentiation between the unspecific melting peak at 75.9ºC of negative samples and the specific melting peak in the E gene assay.

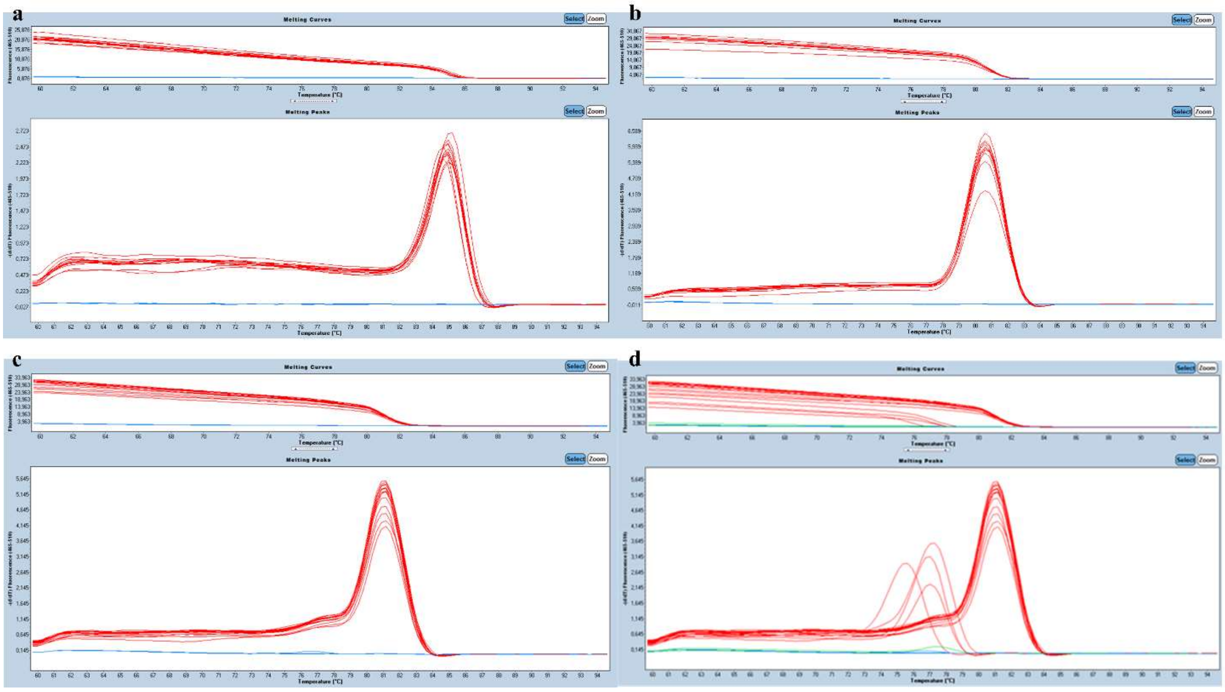

Supplementary figure S2. Limit of detection (LOD) of SYBR RT-qPCR and hydrolysis-probe based RT-qPCR assays.

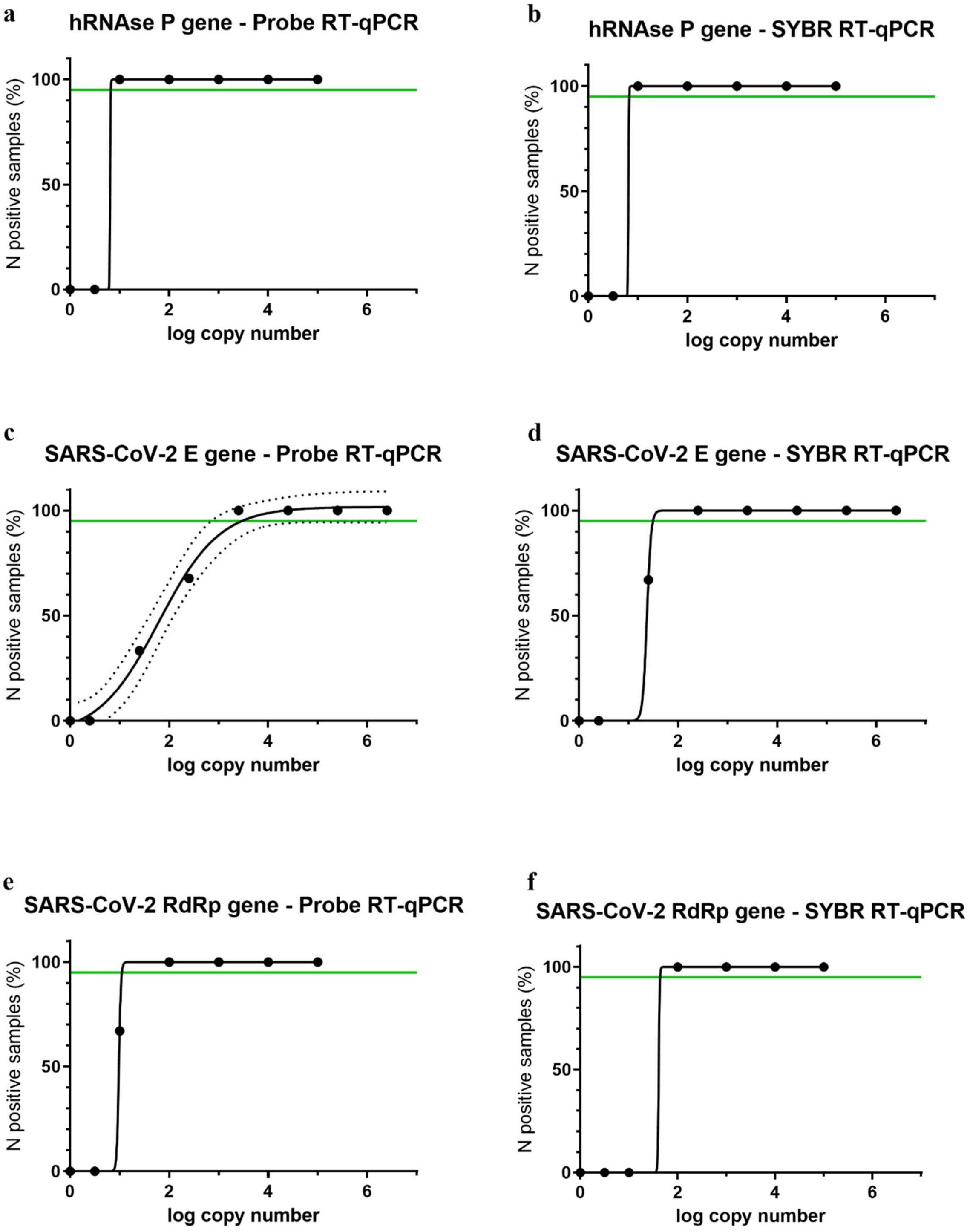

Supplementary table S3. Raw data of non-infected participants (n=6).

	Type of swab	RNA concentration	hRNAse P Probe
		ng/µl	Ct mean
**Classical protocol**	Q-tip	152.2	28.9
		211.2	29.5
		197.5	30.3
		432.8	31.4
		127.8	29.8
		134.0	29.2
	Flocked	16.1	37.5
		23.7	ND
		18.5	ND
		10.1	ND
		26.7	ND
		27.6	ND
	Rayon	32.7	26.5
		13.1	29.0
		25.1	26.2
		12.8	30.4
		110.3	31.2
		40.3	29.9
**QIAamp Viral Mini Kit Protocol**	Q-tip	1.4	30.8
		1.0	31.7
		2.2	30.9
		3.3	29.6
		34.1	27.7
		40.0	26.8
	Flocked	1.0	31.1
		0.4	32.9
		1.14	35.2
		0.87	36.0
		0.37	33.6
		0.48	ND
	Rayon	8.2	26.8
		3.3	28.4
		1.44	29.0
		1.97	30.6
		2.29	27.6
		2.14	30.0

ND: Not detected

Supplementary table S4. Raw data of infected participants (n=7).

	Type of swab	RNA concentration	hRNAse P gene			SARS-CoV-2 E gene			SARS-CoV-2 RdRp gene		
			Probe	SYBR Green		Probe	SYBR Green		Probe	SYBR Green	
		ng/µl	Ct mean	Ct mean	Tm mean	Ct mean	Ct mean	Tm mean	Ct mean	Ct mean	Tm mean
Classical Protocol	Q-tip	41.6	26.3	26.1	84.7	32.8	33.0	80.1	ND	ND	ND
		61.9	26.1	26.1	84.0	ND	ND	ND	ND	ND	ND
		96.2	23.9	23.9	84.3	29.0	29.5	80.0	32.7	30.6	80.6
		27.2	24.4	24.4	84.6	29.3	29.3	80.3	30.7	32	80.4
		23.2	24.7	24.5	84.3	33.1	33.5	80.6	ND	ND	ND
		57.1	26.5	26.5	84.7	ND	ND	ND	ND	ND	ND
		45.7	26.7	26.8	84.0	ND	ND	ND	ND	ND	ND
	Flocked	73.79	34.3	35.3	84.7	35.3	35.4	80.1	33.1	35.0	80.2
		11.87	36.6	36.4	84.8	ND	ND	ND	ND	ND	ND
		80.96	32.8	33.2	84.4	33.7	30.1	80.1	34.3	31.7	80.5
		57.26	29.5	29.6	84.3	32.2	31.4	79.9	33.4	31.2	80.4
		1.2	28.7	29.0	84.7	31.0	30.3	80.6	31.8	30.8	80.3
		10.6	30.1	29.9	84.5	ND	ND	ND	ND	ND	ND
		9.8	31.9	31.9	84.6	ND	ND	ND	ND	ND	ND
	Rayon	23.1	28.3	29.3	84.3	30.8	29.3	80.4	30.6	30.4	80.2
		39.8	27.5	27.7	84.5	31.9	33.3	80.3	33.3	32.1	80.5
		84.9	24.8	24.7	84.4	27.5	27.7	80.0	31.5	28.0	80.5
		18.9	26.2	26.3	84.6	31.1	30.9	80.5	31.6	ND	ND
		21.9	26.2	26.1	84.5	32.8	33	80.6	33.2	32.9	80
		45.3	28.4	28.3	84.9	ND	ND	ND	ND	ND	ND
		51.0	29.0	29.1	84.8	ND	ND	ND	ND	ND	ND
QIAamp Viral Mini Kit Protocol	Q-tip	80.3	25.1	25.0	84.7	31.7	31.9	80.7	35.1	ND	ND
		80.8	23.9	24.0	84.8	28.8	29.0	80.3	34.1	ND	ND
		28.0	31.8	31.2	85.0	39.0	38.0	80.6	ND	ND	ND
		46.0	31.6	31.1	84.8	28.0	28.0	80.5	ND	27.5	79.9
		68.3	25.8	24.7	84.5	29.1	28.7	80.5	35.0	31.3	79.8
		35.9	24.3	24.3	84.9	ND	ND	ND	ND	ND	ND
		67.9	27.8	27.8	84.8	33.5	32.8	80.6	ND	ND	ND
	Flocked	42.1	31.5	ND	ND	33.7	ND	ND	35.5	ND	ND
		36.8	31.4	ND	ND	33.6	ND	ND	35.6	ND	ND
		63.6	27.6	27.0	84.9	31.2	30.6	80.5	34.2	33.0	80.5
		68.7	28.0	28.2	84.7	31.5	31.4	80.7	33.9	31.6	80.6
		74.3	30.1	29.8	84.8	33.0	33.2	80.5	38.7	34.5	80.5
		67.5	31.9	31.8	84.7	ND	ND	ND	ND	ND	ND
		64.7	29.2	28.5	84.7	30.3	29.9	80.5	35.1	30.8	80.0
	Rayon	69.1	25.9	25.1	84.6	29.6	28.3	80.6	32.6	30.9	80.6
		64.7	29.6	ND	ND	ND	ND	ND	ND	ND	ND
		39.3	28.4	28.2	84.8	31.1	32.2	80.6	35.0	31.1	80.7
		61.6	29.0	28.9	84.8	33.5	35.2	80.5	37.5	34.4	80.5
		52.6	26.9	26.3	84.9	27.8	27.7	80.5	33.0	31.7	80.1
		58.6	26.8	26.6	84.7	ND	ND	ND	ND	ND	ND
		43.5	25.9	25.6	84.9	31.5	31.4	80.6	ND	ND	ND

ND: Not detected
